# Validation of the 3-variable prognostic score (3-PS) in mCRPC patients treated with ^223^Radium-dichloride: a national multicenter study

**DOI:** 10.1007/s12149-020-01501-7

**Published:** 2020-07-11

**Authors:** Viviana Frantellizzi, Fabio Monari, Manlio Mascia, Renato Costa, Giuseppe Rubini, Angela Spanu, Arianna Di Rocco, Elisa Lodi Rizzini, Luca Cindolo, Maria Licari, Valentina Lavelli, Susanna Nuvoli, Cristina De Angelis, Valeria Dionisi, Cristina Ferrari, Giuseppe De Vincentis

**Affiliations:** 1 Department of Molecular Medicine, “Sapienza” University of Rome, Viale Regina Elena 324, 00161 Rome, Italy; 2 Radiation Oncology Center, S. Orsola-Malpighi Hospital, Bologna, Italy; 3 Unit of Nuclear Medicine, “Spirito Santo” Hospital, Pescara, Italy; 4 Unit of Nuclear Medicine, Biomedical Department of Internal and Specialist Medicine, University of Palermo, Palermo, Italy; 5 Nuclear Medicine Department, University of Bari “Aldo Moro”, Bari, Italy; 6 Unit of Nuclear Medicine, Department of Medical, Surgical and Experimental Sciences, University of Sassari, Sassari, Italy; 7 Department of Public Health and Infectious Diseases, “Sapienza” University of Rome, Rome, Italy; 8 Nuclear Medicine Unit, S. Orsola-Malpighi Hospital, Bologna, Italy; 9 Department of Urology, “Villa Stuart” Private Hospital, Rome, Italy; 10 Department of Radiological Sciences, Oncology and Anatomical Pathology, “Sapienza” University of Rome, Rome, Italy

**Keywords:** ^223^Radium-dichloride, mCRPC, Overall survival, Prognostic score

## Abstract

**Objective:**

Radium-223 (^223^Ra) has been approved for treatment in patients with metastatic castration-resistant prostatic cancer (mCRPC) and bone metastasis. This α-emitting radionuclide has a beneficial effect on pain and is also capable to increase overall survival (OS). Several studies evaluated the prognostic value of different biomarkers at baseline, such as serum values, imaging parameters or pain. To date, however, clinicians lack a validated and simple system to assess which patients will most likely benefit from ^223^Ra treatment. The 3-variable prognostic score (3-PS), proposed in a single-center study in 2017 classifies patients in five prognostic groups with a specific OS. This study aims to validate the 3-PS in a larger multicenter population.

**Methods:**

Four hundred and thirty mCRPC patients treated with ^223^Ra from six different centers were analyzed. The 3-PS score consists of the collection of baseline hemoglobin, prostatic specific antigen and Eastern cooperative oncology group performance status and was initially applied to the whole population (total group). The score was then validated on the 338 patient’s subgroup (clean group) obtained by subtracting the 92 patients enrolled for the original study of the 3-PS score. This purified group served as further validation evidence.

**Results:**

Statistical analysis showed that the 3-PS score was valid on the total group as well as in the clean group as the AUC estimated (0.74) falls within the CI of the AUC calculated on the validation sample (95% CI 0.66–0.82).

**Conclusion:**

This study confirms the validity of the 3-PS score for mCRPC patients. This score is simple, noninvasive and affordable and can be easily used to select patients that will most probably complete ^223^Ra treatment. In addition, this tool provides an exact estimate of life expectancy in terms of OS.

## Introduction

From its first validation in 2013 in the ALSYMPCA trial [1] radium 223-dichloride (^223^Ra) has been routinely used as a palliative treatment in patients with metastatic castration-resistant prostate cancer (mCRPC) and symptomatic bone metastasis [2]. Two other radionuclides have already been used for their palliative effect on skeletal pain, ^153^Samarium and ^89^Strontium. However, unlike these two radioisotopes, ^223^Ra showed not only a clinical benefit on pain but also a curative effect, prolonging overall survival (OS). Over time, several biomarkers have been tried to unequivocally predict OS or to predict which patients will likely benefit from ^223^Ra therapy and which will likely develop adverse effects (AEs) or bone marrow toxicity [3, 4]. To date, however, no one has proven to be a reliable predictor of the OS. Various attempts have been made using, for example, prostatic specific antigen (PSA) or total alkaline phosphatase (tALP). Unfortunately, PSA could show a “flare phenomenon” [5–7] after the beginning of ^223^Ra treatment that does not necessarily indicate a worsening of the patients’ condition. On the other hand, tALP along with lactate dehydrogenase (LDH) could be used to follow the response to ^223^Ra but it does not correlate with OS [8]. A validated tool that takes into consideration patients’ characteristics and biomarkers is needed to predict OS allowing a more careful selection of eligible patients who would most benefit from ^223^Ra treatment. A retrospective study [9] collected data from 92 patients from a single center and identified a 3-variable prognostic score (3-PS) to predict OS accurately in patients with mCRPC under treatment with ^223^Ra. This score includes baseline Eastern cooperative oncology group performance status (ECOG PS), hemoglobin (Hb) and PSA. This multicenter study aims to evaluate and validate the 3-PS on a larger cohort to provide an effective and accessible tool that could be easily used to determine the suitability of mCRPC patients for ^223^Ra.

## Materials and methods

### Study cohort (total group)

A total of 430 consecutive patients from six different Nuclear Medicine centers with CRPC and symptomatic bone metastasis under treatment with ^223^Ra were included. Therapy with ^223^Ra was conducted in accordance with the European guidelines [10]. Briefly, six intravenous injections (55 kBq per kg of body weight) administered every 28 days in patients with at least six bone metastasis and without known visceral metastasis verified with a CT scan. The burden of skeletal disease was defined through a ^99m^Tc-diphosphonate bone scan performed before the first administration of ^223^Ra and expressed in accordance with the Soloway classification [11]. Patients included were mCRPC males of > 18 years of age with symptomatic bone metastasis. mCRPC is defined by disease progression despite androgen-deprivation therapy [12], with testosterone serum levels < 50 ng/ml and with one or any combination of a continuous rise in serum levels of PSA, progression of pre-existing disease or appearance of new metastases [13]. Patients were considered eligible for ^223^Ra treatment (from 2018) if they already received at least two other lines of systemic therapies for mCRPC or if they could not receive alternative systemic treatment. We enrolled subjects starting from July 2015. Exclusion criteria were represented by impaired kidney and liver function and inflammatory bowel disease (IBD). Serum levels of Hb, PSA and tALP along with red blood cell count, neutrophils count, platelets count (PLT) and LDH were obtained at baseline, after every administration of ^223^Ra and at 3 and 6 months after the last administration in the follow-up. Moreover, baseline age, height, weight, BMI, Gleason score, ECOG PS and number of previous systemic treatments were retrospectively obtained for every patient.

### Validation cohort (clean group)

A subset of patients is extracted excluding those patients already enrolled in the original 3-PS study (*n* = 92) [9]. The clean group consisting of 338 patients, was analyzed for the statistical validation of the 3-PS.

The 3-PS scoring system is shown in Table 1.

**Table 1:** The 3-variable predictive score scoring system

	3-Variable predictive score
Baseline ECOG PS	
0 1 ≥ 2	0 1 2
Baseline PSA	
< 20 ng/ml ≥ 20 ng/ml	0 1
Baseline Hb	
≥ 12 g/dl < 12 g/dl	0 1

This multicenter retrospective study was formerly authorized by the local Ethical Committee and was conducted in conformity with the Declaration of Helsinki and successive amendments. All the participants gave their informed consent to take part in the study.

### Statistical methods

Data are expressed as mean ± standard deviation or median ± IQR as appropriate. OS was defined as the time elapsed from the first administration of 223 Ra until death from any cause or censoring at the last follow-up time. The Kaplan–Meier estimator was used to estimate survival curves. Univariate analysis using a Cox regression model was used to assess potential prognostic factors. A multivariable Cox regression model was then estimated where the final set of predictors was selected based on minimization of the Akaike information criterion (AIC) in stepwise selection stages.

The prognostic significance of the scores was evaluated via time-dependent receiver operating characteristic (ROC) curves in the clean group. Area under the curve (AUC) was also estimated and their significance was assessed via the bootstrap.

The findings were validated by checking that the area under the receiver operating characteristic curve (AUROC) was not significantly different from the results of the original study [6]. All tests are two-tailed, a *p* < 0.05 was deemed as statistically significant. All analyses were carried out using R version 3.6.1.

## Results

**Fig. 1:**
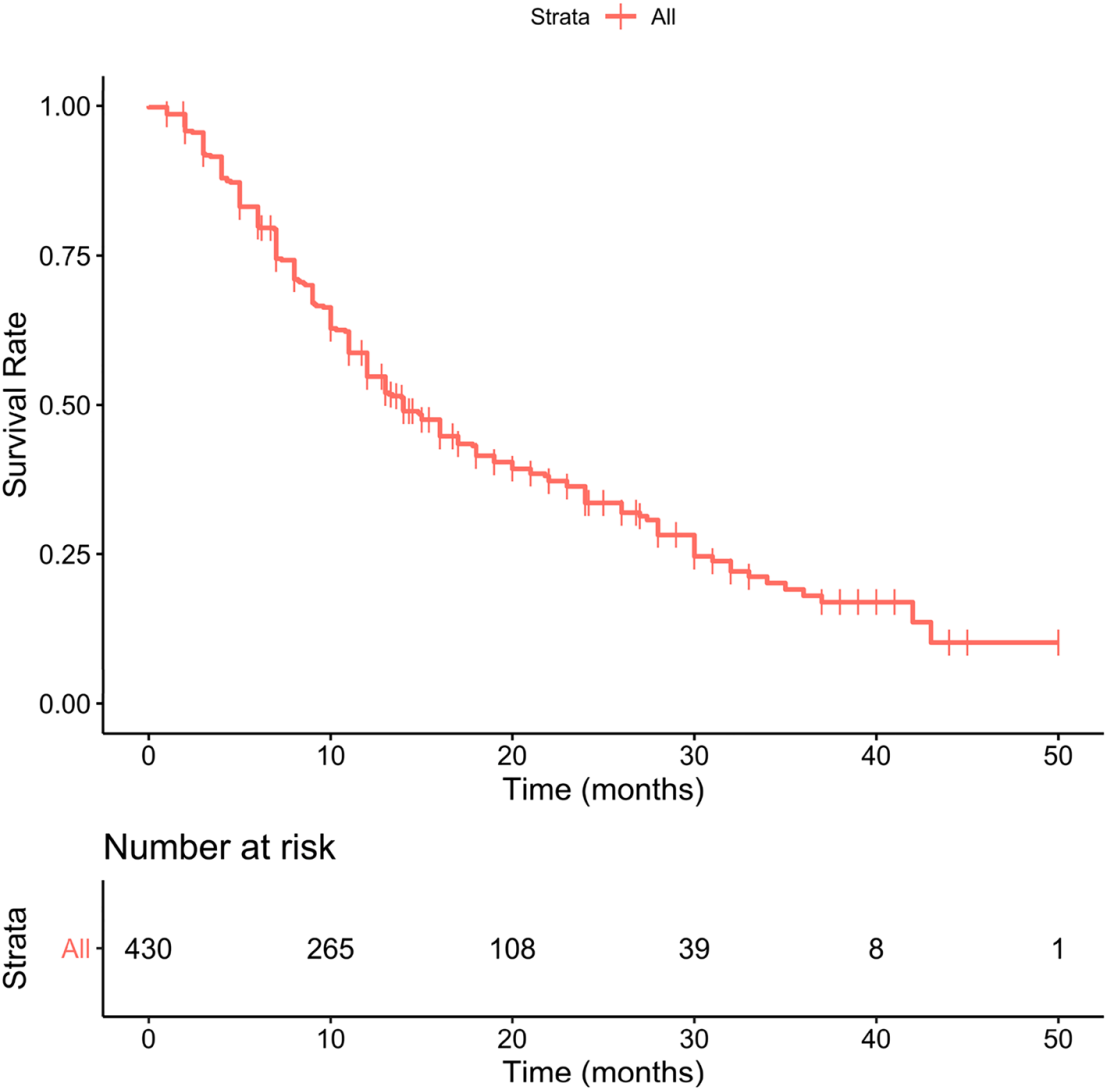
The Kaplan–Meier estimate show OS in the total group

**Fig. 2:**
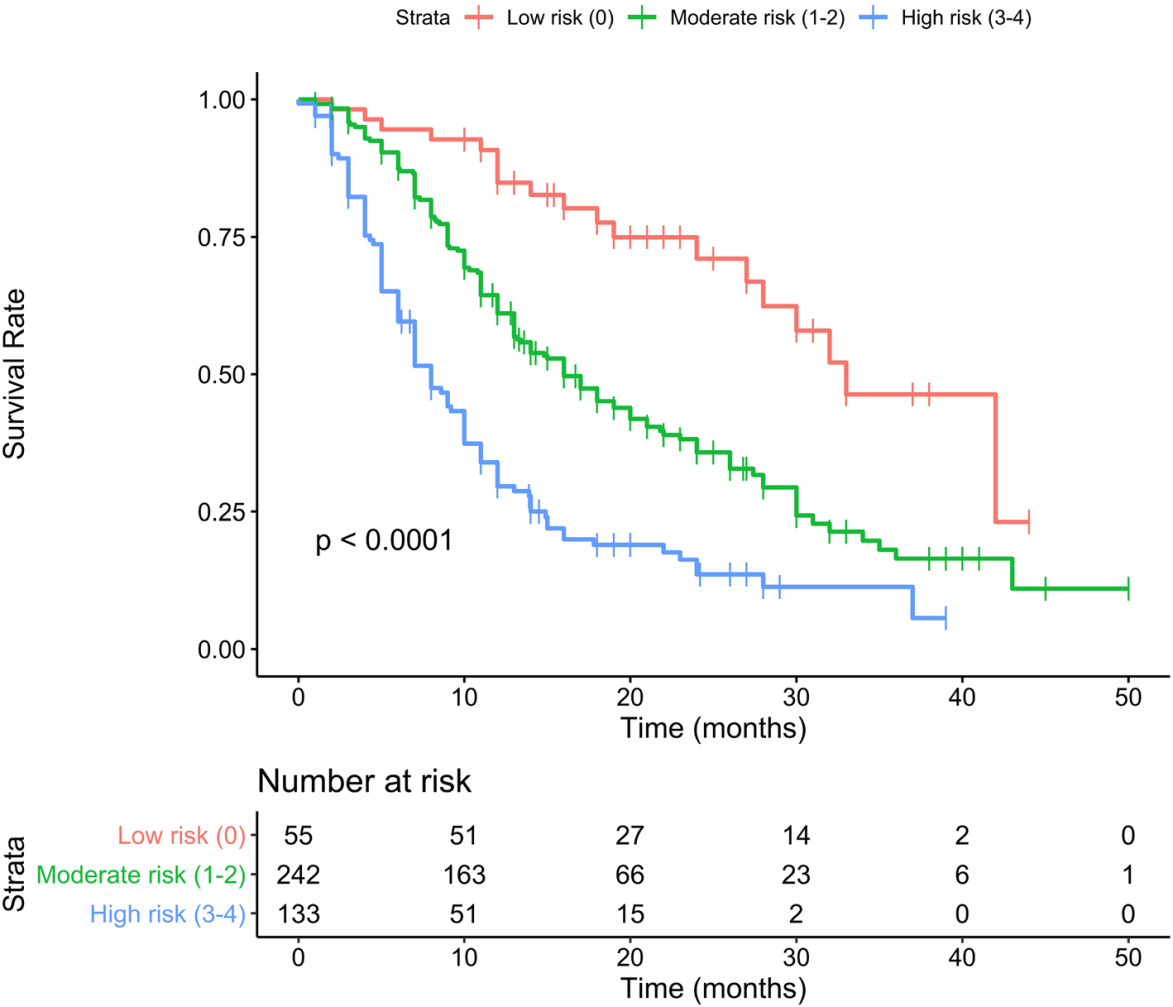
Kaplan–Meier estimate showing the total group layered in five prognostic groups based on OS

**Fig. 3:**
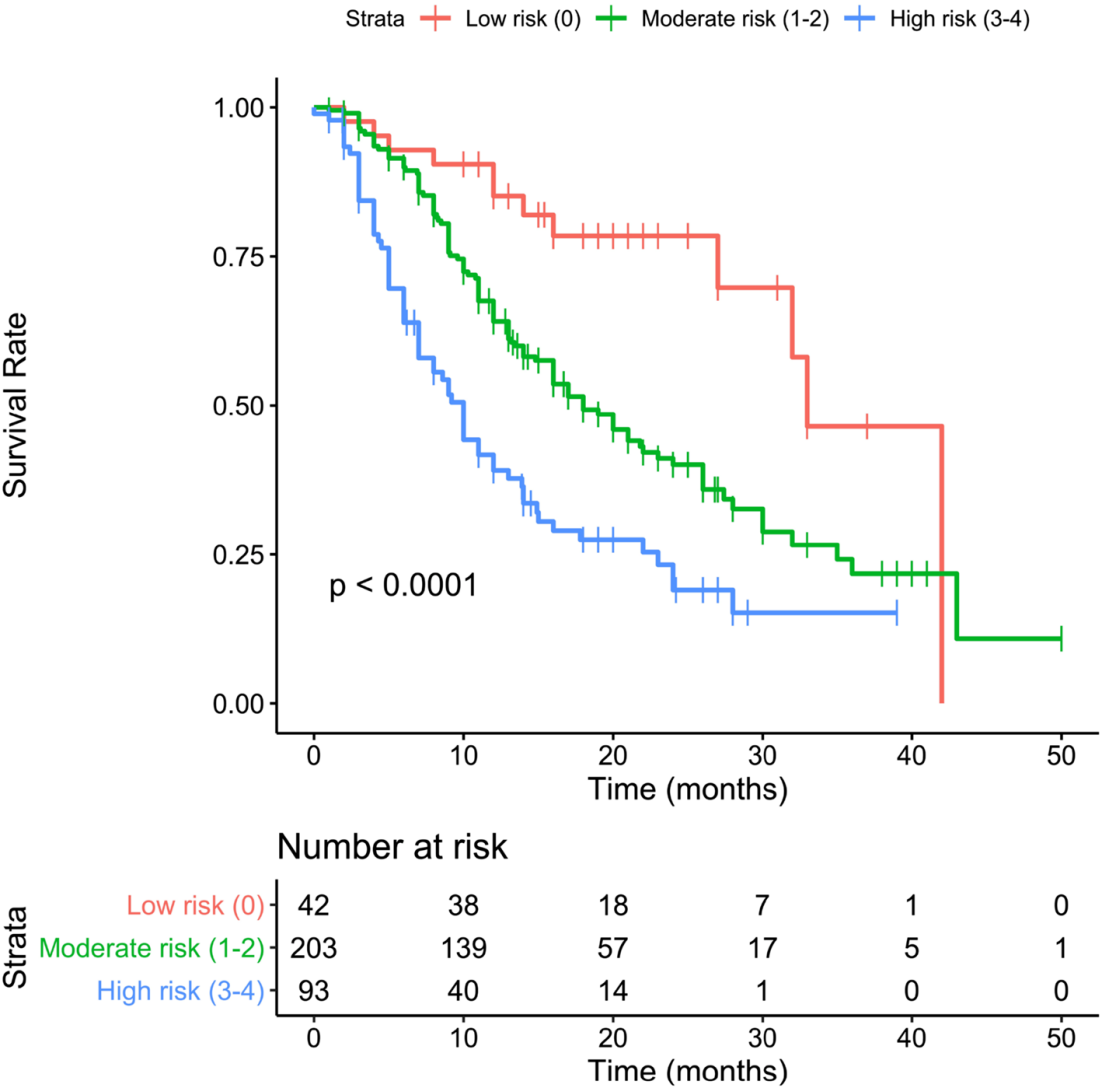
Kaplan–Meier estimate showing the clean group layered in five prognostic groups based on OS

The total group consisted of 430 patients while the clean group consisted of 338 patients. All Patients’ baseline characteristics are shown in detail in Table 2, comprehending both the group of 430 (total group) and the group of 338 subjects (clean group). Considering the total group, the mean age was 74.1 years (50–92) with a mean BMI of 26.8 (14.9–46.2) and a median ECOG of 0. The median Gleason score was 9. For 65 patients ^223^Ra was the first-line treatment while 117, 74 and 78 subjects, respectively, received 1, 2 and more than 3 lines of systemic therapy. 98 patients had < 6 metastases, 266 had between 6 and 20 metastasis and 66 subjects had more than 20. Median baseline Hb was 12.3 g/dl (9.6–15.9) and median baseline tALP was 145 U/l. 140 patients had a baseline PSA value of < 20 ng/ml while 290 subjects had more than 20 ng/ml of PSA value. 265 patients (61.6%) received six cycles of ^223^Ra. 44 patients received five cycles of ^223^Ra, 36 patients four cycles, 33 three cycles, 27 two cycles and 27 patients one cycle. Taking into consideration the prognostic value of all baseline clinical variables, the univariate analysis showed that in the clean group, patients’ ECOG PS, PSA, tALP and Hb values were independently associated with OS. On multivariate analysis, only ECOG PS and Hb values remained significantly correlated with OS. On the other hand, in the total group in addition to ECOG PS, PSA, tALP and Hb values also the number of systemic treatments received after castration and before ^223^Ra therapy was independently associated with OS. However, this variable did not survive on multivariate analysis. Univariate e multivariate analyses are shown in Table 3. Following the original 3-PS score, we divided both total and clean patients into 3 prognostic groups. Each subgroup has a different OS as reported separately for both groups in Tables 4 and 5. Median overall survival time for the entire population was 14 months (95% CI 13–17 months), as shown in Fig. 1. Two others Kaplan–Meier estimates were built, based on the 3-PS scores, obtaining populations with different risk classes and stratifying separately the population of the total group (Fig. 2) and the clean group (Fig. 3). The 3-PS score was validated because the estimated AUC (0.74) falls within the CI of the AUC calculated on the validation sample (clean group) [95% CI 0.66—0.82].

**Table 2:** Baseline patients’ characteristics

Baseline variable	Total group (*n* = 430)	%	Clean group (*n* = 338)	%
Age(years)				
Mean (range)	74.1 (50–92)		74.3 (51–92)	
Height (m)				
Mean (range)	1.71 (1.56–1.95)		1.71 (1.56–1.89)	
Weight (kg)				
Mean (range)	78.3 (48–140)		78.5 (48–140)	
BMI				
Mean (range)	26.8 (14.9–46.2)		26.9 (14.9–46.2)	
Gleason score				
Mean (range)	8 (5–10)		8 (5–10)	
5	3	0.7	3	0.9
6	22	5.1	20	5.9
7	108	25.1	86	25.4
8	99	23.0	77	22.8
9	110	25.6	87	25.7
10	9	2.1	9	2.7
Unknown	79	18.4	56	16.6
ECOG performance status				
Mean (range)	0.8 (0–3)		0.8 (0–3)	
0	181	42.1	148	43.8
1	152	35.3	117	34.6
≥ 2	97	22.6	73	21.6
No. of previous systemic treatment				
0	65	15.1	45	13.3
1	117	27.2	87	25.7
2	74	17.2	50	14.8
≥ 3	78	18.1	60	17.8
Skeletal burden				
≤ 6 mets	98	22.8	88	26.0
6–20 mets	266	61.9	193	57.1
≥ 20 mets	66	15.3	57	16.9
Baseline Hb				
Median (range)	12.3 (9.6–15.9)		12.4 (9.6–15.9)	
< 12 g/dl	177	41.2	131	38.8
≥ 12 g/dl	253	58.8	207	61.2
Baseline tALP				
Median (range)	145 (11.6–1798.0)		130 (11.6–1798.0)	
< 226 U/l	295	68.6	247	73.1
≥ 226 U/l	135	31.4	91	26.9
Baseline PSA				
< 20 ng/ml	140	32.6	115	34.0
≥ 20 ng/ml	290	67.4	223	66.0

**Table 3:** Univariate and multivariable analysis of OS in relation to baseline variables in the TOTAL and clean group

Clinical covariates	Total group univariate models HR (95% CI)	*p* value	Total group multivariable model HR (95% CI)	*p* value	Clean group univariate models HR (95% CI)	*p* value	Clean group multivariable models HR (95%)	*p* value
Age	1.007 (0.992–1.023)	0.371			1.008 (0.990–1.027)	0.369		
BMI	0.948 (0.918–0.979)	0.001			0.952 (0.917–0.990)	0.012		
Gleason score	0.928 (0.818–1.053)	0.246			0.945 (0.818–1.092)	0.445		
ECOG performance status	1.534 (1.325–1.775)	< 0.001	1.486 (1.282–1.723)	< 0.001	1.398 (1.166–1.676)	< 0.001	1.345 (1.121–1.615)	0.001
No. of previous systemic treatments	1.227 (1.098–1.371)	< 0.001			1.212 (1.058–1.389)	0.005		
Baseline Hb	0.722 (0.663–0.787)	< 0.001	0.729 (0.669–0.795)	< 0.001	0.709 (0.636–0.787)	< 0.001	0.717 (0.643–0.799)	< 0.001
Baseline PSA	1.001 (1.001–1.001)	< 0.001			1.001 (1.001–1.001)	< 0.001		
Baseline tALP	1.001 (1.001–1.002)	< 0.001			1.002 (1.001–1.002)	< 0.001

**Table 4:** OS subgroup analysis with 3-PS cohort stratification: total group

Score	Number at risk	Events	OS months, median	LCL 95%	UCL 95%
Low risk (score 0)	55	19	33	28.0	NA
Moderate risk (score 1–2)	242	149	16	13.2	20.0
High risk (score 3–4)	133	105	8	7.0	10.0

**Table 5:** OS subgroup analysis with 3-PS cohort stratification: clean group

Score	Number at risk	Events	OS months, median	LCL 95%	UCL 95%
Low risk (score 0)	42	12	33	32.0	NA
Moderate risk (score 1–2)	203	112	16	16.0	23.0
High risk (score 3–4)	93	65	8	7.0	13.0

## Discussion

### Baseline characteristics: ECOG and CTC

Since the introduction of ^223^Ra in clinical practice, several attempts have tried to identify variables or serologic values that correlated with OS to better select eligible patients. Many studies have therefore been conducted to find prognostic factors for OS. A retrospective analysis published in June 2019 showed that patients with an ECOG PS < 2 had significantly longer median OS (10 months) when compared with patients with an ECOG PS ≥ 2. Moreover, concomitant treatment with abiraterone or enzalutamide did not increase the risk of toxicity [14]. A phase II multicenter trial demonstrated that patients with a baseline ECOG PS ≥ 2, ≥ 3 lines of previous systemic treatment and lower baseline Hb values were less likely to complete all six cycles and consequently less likely to benefit from ^223^Ra therapy. Furthermore, as in the study aforementioned patients tolerated ^223^Ra treatment independently from prior or concomitant therapy with abiraterone or enzalutamide [15]. In this context patients’ general condition can be defined through baseline ECOG PS, PSA, and Hb value. In a broader view, every mCRPC patient’s status could be assessed before not only ^223^Ra but, more in general, before any kind of systemic treatment [16, 17]. In this way, we could choose better patients that will most likely benefit from a sooner administration of ^223^Ra therapy. More and more studies are demonstrating that patients that benefit the most from ^223^Ra treatment are the ones with better baseline conditions. In fact, these subjects will most likely complete all 6 cycles with a higher probability of improving their OS. Another interesting perspective translational study conducted on 45 patients considered the value of circulating tumor cells (CTC) count as a prognostic biomarker. The analysis revealed that patients with CTC ≤ 5 /7.5 ml had a higher likelihood of completing treatment with 223Ra. Although data suggest that baseline CTC counts ≤ 5 /7.5 ml also correlates with increased progression-free survival, no statistical significance was achieved [21].

### Toxicity

Two studies [18, 19] take into consideration the potential hematologic toxicity of ^223^Ra. The two papers retrospectively analyzed baseline hematologic parameters to individuate which one could most likely predict ^223^Ra hematologic AEs and thus impairing the completion of all six cycles determining worse OS. Both studies concluded that impaired baseline hematopoiesis (defined by low Hb and low PLTs count) led to a higher probability of AEs and worse OS compared to patients with good Hb levels. However, it is still not clear whether the higher probability of AEs in these subjects depends exclusively on ^223^Ra treatment or previous treatment or the presence of bone metastasis which could have impaired bone marrow functionality. A multicenter study previously published showed that there are no differences in terms of toxicity, safety and OS among less than or more than 75 years old population. The cited work was obtained on the same population sample of this study, however the purpose and results obtained are totally different and have not been duplicated in any way. In fact, while the manuscript presented (3-PS) focuses on the statistical validation of a multidimensional system that allows the better selection of patients who can benefit from ^223^Ra in terms of OS, the previous article published in a geriatric journal, analyzed the population based on age and therefore took into account efficacy, toxicity profiles and survival in elderly patients [20].

### Baseline characteristics: serum markers and imaging

A study published in 2014 used data from a trial of 1901 patients that compared the efficacy of denosumab and zoledronic acid in men with mCRPC [21]. The aim of this study was to confirm the validity of already known prognostic factors and assess the prognostic value of other parameters. The analysis revealed that bone-related parameters such as lower bone-specific alkaline phosphatase (BSAP) levels, lower tALP levels, lower urinary N-telopeptide (uNTx) levels, no history of previous skeletal-related events (SRE), and mild/no pain were predictors of better OS. Two other parameters correlated with OS, namely time since first bone metastasis, and time from the initial diagnosis to first bone metastasis. In addition, the prognostic value of lower PSA levels, absence of visceral metastases, better PS, and higher Hb levels were confirmed. However, these data were not collected with the intent of analyzing them before or during ^223^Ra treatment. An innovative study conducted on 42 patients in 2015 evaluated skeletal tumor burden on whole-body 18F-fluoride PET/CT and correlated it with OS [22]. Two specific indices used to assess skeletal tumor burden namely total fluoride skeletal metastatic lesion uptake (TLF10) and total volume of fluoride avid bone metastases (FTV10) strongly correlated with OS. This study proved that the skeletal tumor burden assessed with baseline 18F-fluoride PET/CT before ^223^Ra treatment was an independent predictor of OS. Moreover, despite the heterogeneous response to ^223^Ra treatment statistical analysis showed that OS improved accordingly to the number of administrations received.

### Baseline characteristics: pain

A study from 2019 retrospectively analyzed 25 patients with mCRPC treated with ^223^Ra and correlated patient’s basal pain with OS [23]. Basal pain was assessed with the visual analog scale (VAS). The results indicated that a VAS value < 4 significantly correlated with better OS compared to higher VAS values. However, this study presents several limitations: firstly, the small population and secondly the concomitant treatment with systemic drugs such as abiraterone, docetaxel, and enzalutamide that could have altered OS.

### Role of 3-PS

In the present analysis, we considered the 3-PS baseline study conducted in 2017 as a reference for our multicenter evaluation [9]. That retrospective study proposes a simple 3-variable score based on baseline ECOG PS, PSA, and Hb serum levels. The 3-PS has proven to be associated with OS in patients with mCRPC under treatment with ^223^Ra. Using this score, patients can be divided into five prognostic groups with more than 31 months in the first two groups, 11 months, 9 months and 4 months of OS in the remaining three groups, respectively (refer to Table 3 in the same paper). In the starting paper, the AUC value was 78.4% (*p* < 0.001) while in this paper the final AUC value estimated was 74% (*p* < 0.001). The 3-PS score was therefore validated also on this multicentric study because the AUC value was included in the CI (95% CI 0.66–0.82). One of the most innovative characteristics of the 3-PS is that it uses three variables that can be easily obtained before the start of ^223^Ra therapy. However, a limit of the former study is that the population analyzed consisted of only 92 subjects, clearly too small of a sample to validate this score. This limit was exceeded by our multicenter retrospective evaluation using a population composed by 430 patients. This population includes the 92 patients from the previous paper, however we also analyzed separately 338 subjects coming from different centers excluding the patients prior used. In both groups the multivariate analysis showed a statistically significant correlation between ECOG PS, PSA, Hb values at baseline and OS. It is fundamental to understand that it is not important the number of previous treatments received by the patient for OS (see multivariable analysis), but his general clinical status before starting ^223^Ra therapy. Two beneficial effects can be achieved by ^223^Ra treatment: prolonging life expectancy and relieving pain. All patients are potentially suitable when palliation of pain is required, but 3-PS is most useful in patients in whom an increase in OS is desirable and achievable. It is very easy to imagine that the three variables singularly considered are important prognostic factors of OS in several kind of systemic therapies. However, this study has shown that the association of Hb, ECOG PS and PSA levels increases their predictive value in evaluating OS in patients with mCRPC. Indeed, the results of this study confirms that the patients who most benefit from ^223^Ra are those with the higher Hb, lower PSA and better ECOG PS.

## Conclusion

This retrospective multicenter study proved that the 3-variable prognostic score is an easy, valid and reliable tool that can help selecting mCRPC patients at baseline maximizing ^223^Ra beneficial effects. Based on this multidimensional assessment of the patient at the time of enrollment for treatment with ^223^Ra, the clinician may use parameters such as PSA, Hb and ECOG PS which combined together, point towards the best timing to start the most appropriate oncologic therapy.
